# Antithrombogenicity of Fluorinated Diamond-Like Carbon Films Coated Nano Porous Polyethersulfone (PES) Membrane

**DOI:** 10.3390/ma6104309

**Published:** 2013-09-27

**Authors:** Gunawan S. Prihandana, Ippei Sanada, Hikaru Ito, Mayui Noborisaka, Yoshihiko Kanno, Tetsuya Suzuki, Norihisa Miki

**Affiliations:** 1 Department of Mechanical Engineering, Keio University, 3-14-1 Hiyoshi, Kohoku-ku, Yokohama 223-8522, Japan; E-Mails: ippei11@z3.keio.jp (I.S.); h_itoh_0415@z3.keio.jp (H.I.); okura0306@gmail.com (M.N.); tsuzuki@mech.keio.ac.jp (T.S.); miki@mech.keio.ac.jp (N.M.); 2 Department of Nephrology, Tokyo Medical University, 6-7-1 Nishi-Shinjuku, Shinjuku-ku, Tokyo 160-0023, Japan; E-Mail: kannoyh@tokyo-med.ac.jp

**Keywords:** nano porous polyethersulfone, fluorinated diamond-like carbon, blood compatibility, surface modification

## Abstract

A nano porous polyethersulfone (PES) membrane is widely used for aspects of nanofiltration, such as purification, fractionation and dialysis. However, the low-blood-compatibility characteristic of PES membrane causes platelets and blood cells to stick to the surface of the membrane and degrades ions diffusion through membrane, which further limits its application for dialysis systems. In this study, we deposited the fluorinated-diamond-like-carbon (F-DLC) onto the finger like structure layer of the PES membrane. By doing this, we have the F-DLC films coating the membrane surface without sacrificing the membrane permeability. In addition, we examined antithrombogenicity of the F-DLC/PES membranes using a microfluidic device, and experimentally found that F-DLC drastically reduced the amount of blood cells attached to the surface. We have also conducted long-term experiments for 24 days and the diffusion characteristics were found to be deteriorated due to fouling without any surface modification. On the other hand, the membranes coated by F-DLC film gave a consistent diffusion coefficient of ions transfer through a membrane porous. Therefore, F-DLC films can be a great candidate to improve the antithrombogenic characteristics of the membrane surfaces in hemodialysis systems.

## 1. Introduction

Polyethersulfone (PES) membrane is a polymeric type of membrane, which has been used for separation and filtration purposes. PES membrane has an outstanding thermal and mechanical stability and can be fabricated at room temperature. In addition, PES membranes can tolerate many kinds of sterilized methods, including epoxy ethane gas, steam, and γ-ray, and are high permeable to diffuse low molecular weight proteins easily when applied as hemodialysis membranes [1]. Some researchers have employed PES membranes in biomedical applications to make artificial organs, and in the field of medical devices they are mainly used for blood purification purposes: for example, hemodialysis, hemodiafiltration, hemofiltration, plasmapheresis and plasma collection [2,3,4,5].

However, one of the drawbacks of the PES membrane is its low blood compatibility. Therefore, when a PES membrane is used in the blood contact device, proteins from the blood will be absorbed onto the PES membrane surface and will form a protein layer. The formed protein layer on the PES membrane surface may cause platelet adhesion and blood clotting. Consequently, injections of anti-coagulants during medical application are necessary to prevent blood coagulation [6].

In the field of PES membrane modification, there are several conducted works such as bulk modification of PES material [7] and also coating the membrane surface [8] but not many can be applied for the modification of PES membranes. 

In this study, we propose a surface modification of the PES membrane by applying diamond-like carbon (DLC) film. Lately, DLC films have received much attention because of their antithrombogenicity, which prevents platelets adhesion [9,10]. Considering the advantage of antithrombogenicity in preventing the platelets adhesion and it is noted that fluorinate has capability to improve the blood compatibility of materials; therefore we decided to use fluorinated DLC (F-DLC) film as coating material on PES membrane surface. The PES membrane itself has two different parts; one on the top is the diffusion layer which acts as separating part for the ions transport, while the bottom part is called the finger like structure layer. The finger like structure works to reinforce the diffusion layer. In this study, a strategy by depositing the F-DLC film on the finger like structure layer (bottom part of the PES membrane) was taken in order to have the F-DLC film on the membrane surface without losing its permeability. Water contact angle measurements on the membrane surface were performed to confirm the existence of F-DLC films. Moreover, long term diffusion test and blood cells as well as platelet adhesion on the membranes surface were also studied.

## 2. Experimental Section

### 2.1. Materials

Commercial PES (molecular weight: 4800, Sumitomo Chemical Co., Tokyo, Japan) was used as a solute in the membrane fabrication. 1-methyl-2-pyrrolidone (NMP, Wako Pure Chemical Industries, Ltd., Osaka, Japan) and polyvinylpyrrolidone (PVP) (molecular weight: 35,000, Wako Pure Chemical Industries, Ltd., Osaka, Japan) were employed as additive and solvent in the PES solution. Defibrinated bovine blood purchased from Kohjin Bio Co. Ltd. was used as blood solution and NaCl solution as dialysate to follow medical standard. Urea, NaCl (Sodium chloride) and Potassium chloride (KCl) were purchased from Wako Pure Chemical Industries, Ltd., Osaka, Japan. 20% Glutaraldehyde solution and Ethanol were purchased from Wako Pure Chemical Industries, Ltd., Osaka, Japan.

### 2.2. Membrane Fabrication

Flat sheet PES membranes were prepared from PES (molecular weight: 4800, Sumitomo Chemical Co., Tokyo, Japan), polyvinylpyrrolidone (PVP) (molecular weight: 35,000, Wako Pure Chemical Industries, Ltd., Osaka, Japan), and 1-methyl-2-pyrrolidone (NMP, Wako Pure Chemical Industries, Ltd., Osaka, Japan), acting as solute, solvent and additive, respectively. The PES, PVP and NMP were mixed at 20%, 20% and 60% (wt %), respectively, and kept at room temperature for about 48 h to form transparent casting solutions. Subsequently, the PES casting solution was poured onto a glass chip. The PES membrane was then prepared by spin coating at specified spinning speed and then immersed into the distilled water bath to gelatinize the PES solution. As soon as the glass chip sank into the distilled water, a thin layer of white membrane (gelatinized PES solution) could be seen forming at the interface between the casting solution and the distilled water. The thus formed PES membranes were then stored for further use in distilled water at room temperature for more than 24 h to remove the PVP. Pore size formed in the PES membrane is considered to be 2–5 nm [11,12,13].

### 2.3. Membrane Morphology

PES membrane consists of two different layers. The first layer is nanoporous where molecules diffusion and separation is taking a place. The second layer is microporous and it is called as a finger like structure layer; therefore, it has no capability to separate nano size molecules.

The surface morphology and cross section of the membrane were observed by using a Keyence laser microscope VK-X100 and FEI Quanta 200 environmental scanning electron microscope (ESEM). Osmium coater was used to coat the outer surface of the PES membrane with osmium. For cross section analysis, the membrane pieces were frozen using a path freezer (Matsunami, Ltd., Osaka, Japan) and then cut into small pieces. Those small pieces were then kept in air for drying. The dried samples were coated with osmium for producing the electric conductivity. After coating with osmium, the membranes were viewed with ESEM.

### 2.4. Membrane Modification

Fluorinated DLC (F-DLC) films were prepared on a PES membrane using radio frequency (RF) plasma enhanced chemical vapor deposition (CVD) method by changing the ratio of hexafluoroethane (C_2_F_6_) and acetylene (C_2_H_2_). The RF (13.56 MHz) power and total pressure were fixed at 200 W and 13.3 Pa, respectively. F-DLC films were deposited from a mixture of C_2_H_2_ and C_2_F_6_, and the deposition time of F-DLC films were 3 s, 4 s, 5 s and 6 s. The thickness of the F-DLC film produced is in the range of 3.3–6.6 nm, depending on the deposition time. In this study, F-DLC60 was chosen as a deposited film because F-DLC60 has high concentration of fluorine (50% of F) with relatively less hydrophobic surface than other types of F-DLC [10]. According to partial pressure of C_2_F_6_, F-DLC films were denoted as F-DLC60 indicates that F-DLC films were deposited under partial pressure of C_2_F_6_ at 60% of the total pressure, where the composition of F-DLC60 film is 45% of C, 5% of O and 50% of F. F-DLC films were then deposited on the PES membrane surface.

### 2.5. Contact Angle Measurement

In order to examine variations in the hydrophobicity of pure and modified PES membrane, as a function of FDLC thickness layer, water contact angle measurements of PES membranes were conducted using contact angle measuring instrument (DM 500, Kyowa Interface Science Co. Ltd., Saitama, Japan). This measurement represents the surface wetting characteristic of the membrane. Deionized water was used as the probe liquid. The contact angle was measured in at least three random locations for each sample and the average calculated. 

### 2.6. Diffusion Test Experiment

The diffusion test was performed to evaluate the permeability and biocompatibility of the membrane before and after F-DLC deposition. The tests were conducted in two different types of diffusion, short term and long term diffusion tests. The short term diffusion test (3 h) was used to examine the permeability of the membranes before and after F-DLC deposition process and evaluate the membrane permeability for long term diffusion test. On the other hand, a long term diffusion test was conducted for 24 days in order to study the blood compatibility and performance of the membrane during contact with blood solution. 

**Figure 1:**
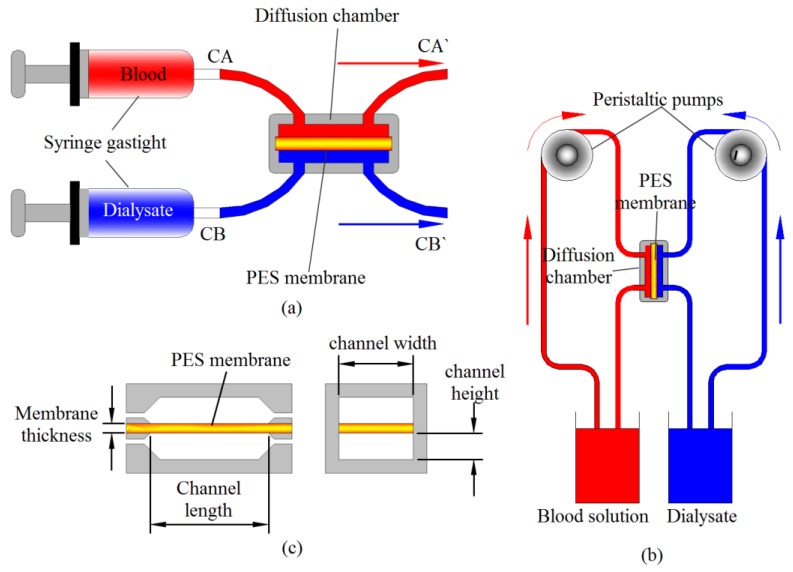
Diffusion test set up: (**a**) short term diffusion test setup; (**b**) long term diffusion test setup; (**c**) parameters used in diffusion coefficient measurement.

In the short term diffusion test, the diffusion chamber was made by a Polymethylmethacrylate plate. The tested membrane was clamped between the blood and dialysate inside the diffusion chamber. Gastight syringes (Hamilton Company, Reno, NV, USA) were used to flow the blood and dialysate to the diffusion chamber, as illustrated in Figure 1a. The blood was flowed on the finger like structure layer of the membrane, which was coated by F-DLC film. Due to the micro-porosity properties of the finger like structure layer, which may cause blood leakage during the diffusion test, the Polydimethylsiloxane was casted on the edges of the membrane in order to seal the leakage. The short term diffusion test was also used to calculate diffusion coefficient of membrane on the specified measurement day during the long term diffusion experiment. 

For the long term diffusion test, a loop system representing a portable dialysis system was designed and built, as illustrated in Figure 1b. In this system, a peristaltic pump (Peri-Star Pro, World Precision Instrument) was used to circulate the blood and dialysate into the diffusion chamber. On the measurement day, the chamber was disconnected from the loop system (long term diffusion set up) and then plugged it into the short term diffusion test system for permeability evaluation purpose. The same method (short term diffusion test) to evaluate the membrane permeability warrants a valid result of diffusion coefficient measurement.

In this diffusion test, blood and dialysate solution were used as fluid medium for solute transport. Defibrinated bovine blood was used as a blood solution and a NaCl and K solution, which follows the medical standard, was used as dialysate solution. Urea was added to the blood to achieve concentration of blood urea nitrogen (BUN) to 100 mg/dL. The concentrations of Na, K and Cl in blood and dialysate were measured using an electrolyte analyzer (SPOTCHEM EZ SP-4430, ARKRAY, Inc., Kyoto, Japan), whilst, the urea concentration was measured using automated analyzer for clinical chemistry (SPOTCHEM EZ SP-4430, ARKRAY, Inc., Kyoto, Japan). The detail concentrations of the defibrinated bovine blood and dialysate solution are presented in Table 1.

**Table 1:** Urea, Na, K and Cl concentrations in defibrinated bovine blood and dialysate.

Solution	Urea (mg/dL)	Na (mmol/L)	K (mmol/L)	Cl (mmol/L)
Defibrinated bovine blood	100	128	4.4	89
Dialysate	<5	141	2.3	88

Table 2 shows the electrolyte values in the dialysate before and after the diffusion test during preliminary experiments. In the preliminary experiments, Na^+^, Cl^−^ and K^+^ ions were used as representative molecules smaller than 0.5 nm. Among those ions, only the K^+^ presented significant decreases after the diffusion test, whereas Na^+^ and Cl^−^ did not change. The accurate K^+^ values are critically important for the management of patients with little or no residual kidney function [14,15]. Therefore, in the further diffusion test, the changing concentration of K^+^ in blood and dialysate were used to determine the permeability of the membrane.

The methodology for calculating diffusion coefficient of the membrane has been described previously [12,13]. In current study, some modifications were made and the diffusion coefficient was determined based on the following equation:

(1) $$D_{c}=\frac{Q\times H}{A}\times \ln\left[\frac{C_{B}-C_{A}}{C_{B^{'}}-C_{A^{'}}}\right]$$

where *C_A_* is the initial concentration of K in solution *A* (blood ) and *C_B_* is that in solution B, which is dialysate. *H* is the thickness of the membrane and *A* is the diffusion area of the chamber, as shown in Figure 1c. Diffusion tests were performed in the room temperature (27 °C), where the flow rate of the solution, membrane area and amount of fluid used per one time diffusion test are 5 mL/min, 41.4 mm^2^ and 3 mL respectively. By feeding the blood and dialysate into the dual inlets of the chamber, molecules smaller than the mean pore size of the membrane are able to diffuse through the membrane into the collected solution *B* (*C_B_*_′_). By measuring the concentration of the collected solution *B* and that of solution A (*C_A_*_′_), the diffusion of the solute through the membrane can be obtained. The results of the concentration measurements are expressed as the average of 3 replicates and the corresponding standard deviation.

**Table 2:** Electrolyte values before and after diffusion test on the polyethersulfone (PES) membrane.

Molecules	Pre diffusion test	Post diffusion test
K	148	147
Na	2.3	2.8
Cl	96	93

## 3. Results and Discussion

### 3.1. Membrane Thickness and Membrane Morphology

**Figure 2:**
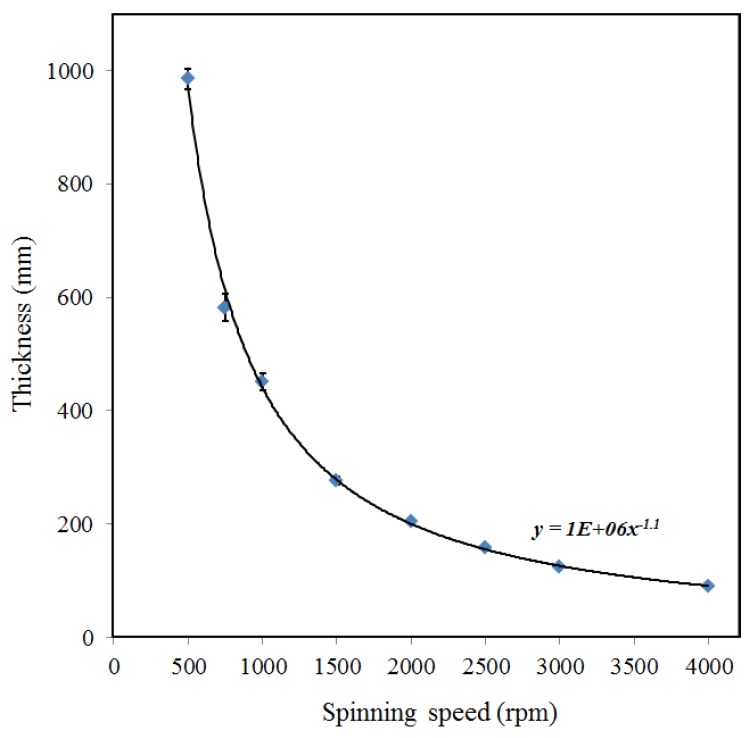
Relationship between spinning speed and PES membrane thickness.

In PES membrane fabrication, spinning speed of the spin coater determines the thickness of the PES membrane. Figure 2 shows the relationship of spin coating speed and membrane thickness. As shown in Figure 2, the spinning speed determines the membrane thickness exponentially. Therefore, the faster the spinning speed, the more thinly the PES membrane will be produced, and vice versa. As can be seen at spinning speed 4000 rpm, the PES membrane thickness produced is around 100 µm, while at relatively low spinning speed which is 1000 rpm, the PES membrane thickness is 420 µm. Therefore, the desired thickness of PES membrane can be determined based on the equation shown in the Figure 2.

**Figure 3:**
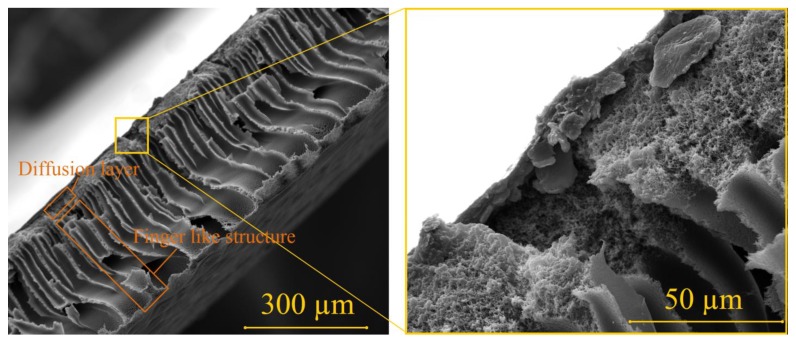
Cross section of PES membrane.

**Figure 4:**
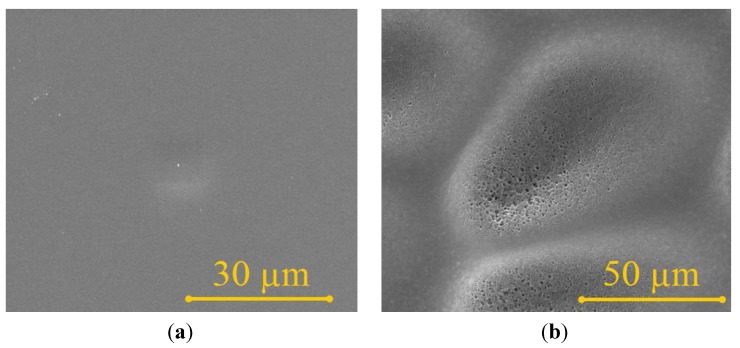
Surface morphology of (**a**) top and (**b**) bottom surface of PES membrane.

PES membrane itself is composed by two different layers, as shown in Figure 3. The first layer acts as a separation for molecules diffusion and the second layer works as to support the diffusion layer, in the form of finger like structure. The first layer has smooth surface (*R*_a_ = 0.7 µm) and it is a nanoporous. The finger like structure layer has micron size porous and the surface roughness is relatively rougher (*R*_a_ = 2.4 µm), as seen in Figure 4. Referring to Figure 2 about the relationship between spinning speed and PES membrane thickness where the lower spinning speed give a thicker membrane, in this case, the thicker membrane has thicker finger like structure layer, while the thickness of diffusion layer remains the same, which is about 70–80 µm of thickness. The finger like structure layer of PES membrane has started to develop at membrane with the thickness is higher than 80 µm. In the filtration process, only the diffusion layer will do the separation process, due to its nanoporous properties. Whilst, the finger like structure layer will only work as a supporter for the thin diffusion layer, due to the bigger porous. The reason behind the formation of this typical structure had been explained in the previous studies [16]. In this study, we aimed to coat F-DLC on the finger like structure layer to avoid the F-DLC films clogs the nanoporous of diffusion layer. Therefore, in order to have the PES membrane composed by finger like structure layer, spinning speed below 2500 rpm is required to have membrane with thickness 100 µm.

### 3.2. Contact Angle Measurement

**Figure 5:**
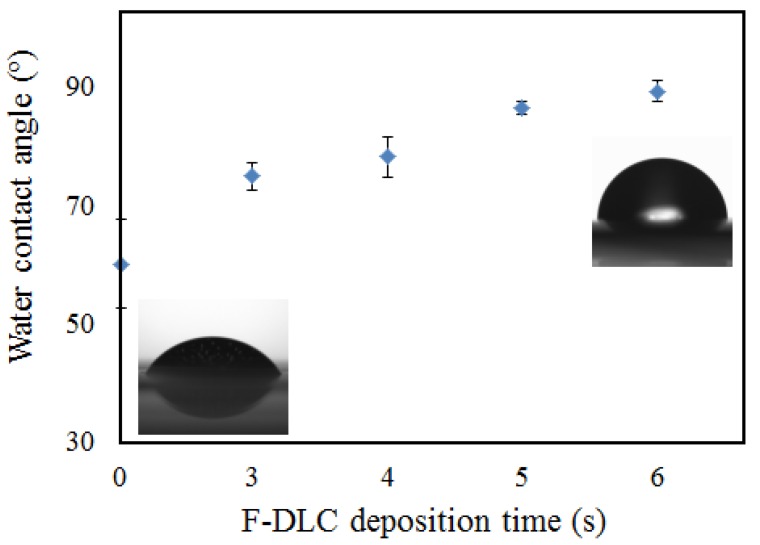
Water contact angle measurement of PES membranes and coated PES membrane.

Contact angle measurements were conducted to evaluate the changes in the hydrophobicity of PES membranes before and after surface modification. Figure 5 shows the membrane surface angles of PES membrane/F-DLC film thickness. Bare PES membrane presented a contact angle of about 60°, corresponding to lower surface hydrophilicity. After coating with F-DLC films, the contact angles increased gradually (F-DLC 3 s (74.9°), F-DLC 4 s (78°), F-DLC 5 s (86.43°), F-DLC 6 s (89°)), which indicated that the modified membranes become hydrophobic with the presence of F-DLC film coating on the surface. The water contact angles of the coated membrane were well within the range with the contact angle of F-DLC film mentioned by Hasebe *et al.* [10]. The results further confirmed that the F-DLC film was successfully coated on the PES membrane surface. 

### 3.3. Blood Compatibility

PES membrane has been widely used in blood contacting devices for the hemodialysis treatment. Therefore it is important to investigate the blood compatibility of PES membrane and PES coated F-DLC film for further used in the biomedical application. In this study, we have conducted the diffusion test on PES membrane and coated PES membrane for 24 days. The evaluated membrane in the short term and long term diffusion test were then characterized using SEM in order to study the membrane blood compatibility.

**Figure 6:**
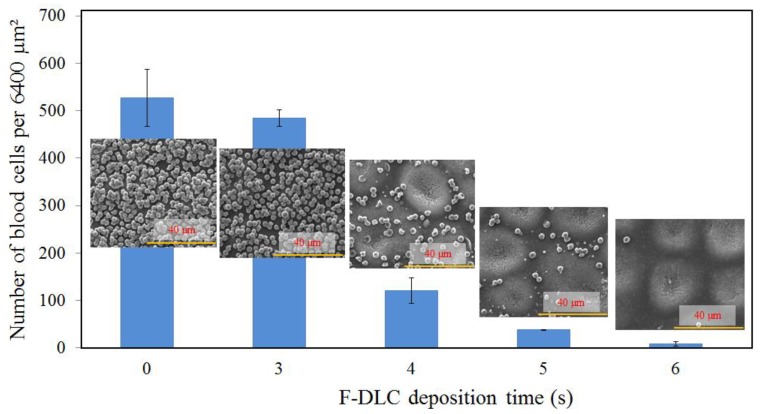
The amount of blood cells adhered on the membrane surface.

Figure 6 shows the scanned electron micrographs of the adhering blood cells on the PES and modified PES membranes in the first day of the diffusion test. For the bare PES membrane, adhered and accumulated blood cells were found. In addition, the number of adhered blood cells in the membrane surface decreased with the increased of F-DLC deposition time. Impressively, almost no blood cell was found on PES membrane coated by F-DLC film with 6 s of deposition time. This is due to the antithrombogenicity characteristic of the F-DLC, which has prevented the blood clot on membrane when the membrane was in contact with the blood. This further confirmed that the introduction of fluorine on the DLC coating has a direct interaction with the blood in improving the blood compatibility of the membrane. Where, the concentration of F and C in the F-DLC60 are 50% and 45% respectively, and the chemical bonding F-DLC60 also show the presence of C–CF bond and C–C bond [10]. The results conclude that the coated F-DLC film on PES membrane have lowered the blood cells adherent and improved the membrane blood compatibility. In addition, PES membrane that has the highest count of blood cells is the most hydrophilic among other membranes.

**Figure 7:**
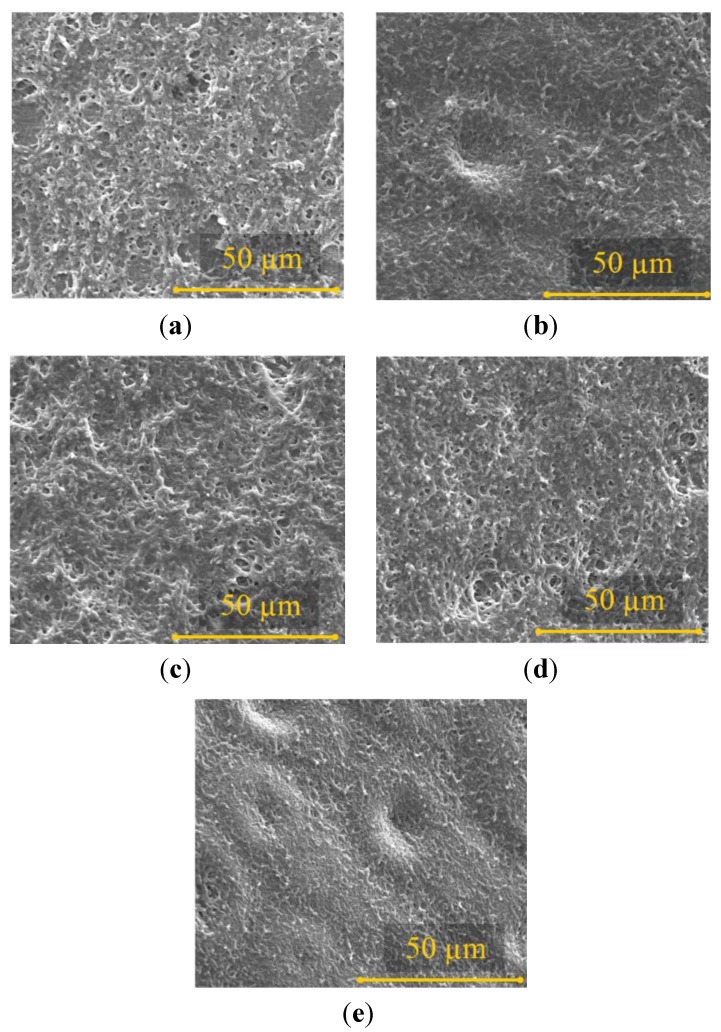
SEM images of the membrane surface after long term diffusion test: (**a**) Bare PES membrane; (**b**) PES/F-DLC 3 s; (**c**) PES/F-DLC 4 s; (**d**) PES/F-DLC 5 s; (**e**) PES/F-DLC 6 s.

The investigation of blood clot and fibrous tissue formation were also conducted on the membrane after diffusion test for 24 days. Figure 7 shows that the adherent of blood clot on the membrane surfaces after 24 days of diffusion test. After 24 days in contact with blood, the bare PES membrane shows some blood clots and tissue formations on its surface. The same condition occurred to the membrane coated by F-DLC films. Apparently, the blood flows in the diffusion test for 24 days has removed the F-DLC film from the membrane surface and the inner porous of the PES membrane. This is probably due to the poor adhesion of the thin F-DLC film on the PES membrane surface and membrane porous. Therefore, the membrane surfaces were fully covered by some blood clots.

Therefore, the membrane surfaces were fully covered by some blood clots. From the result, it can be determined that, the F-DLC films works great to improve the blood compatibility of the membrane in the first day of the diffusion test; however, due to the longer duration of the diffusion test, which was 24 days, the F-DLC films started to peel off from the membrane surface, thus, we found adherent of the blot clots.

### 3.4. Permeability of the PES Membrane after F-DLC Coating

**Figure 8:**
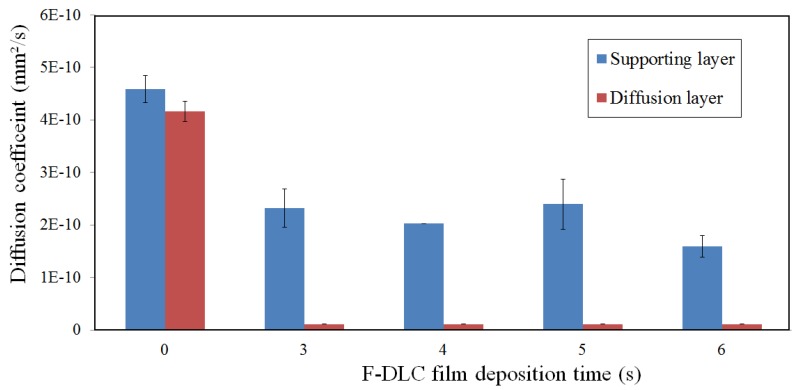
Diffusion coefficients of diffusion and finger like structure layer of PES membrane before and after coated by F-DLC film.

**Figure 9:**
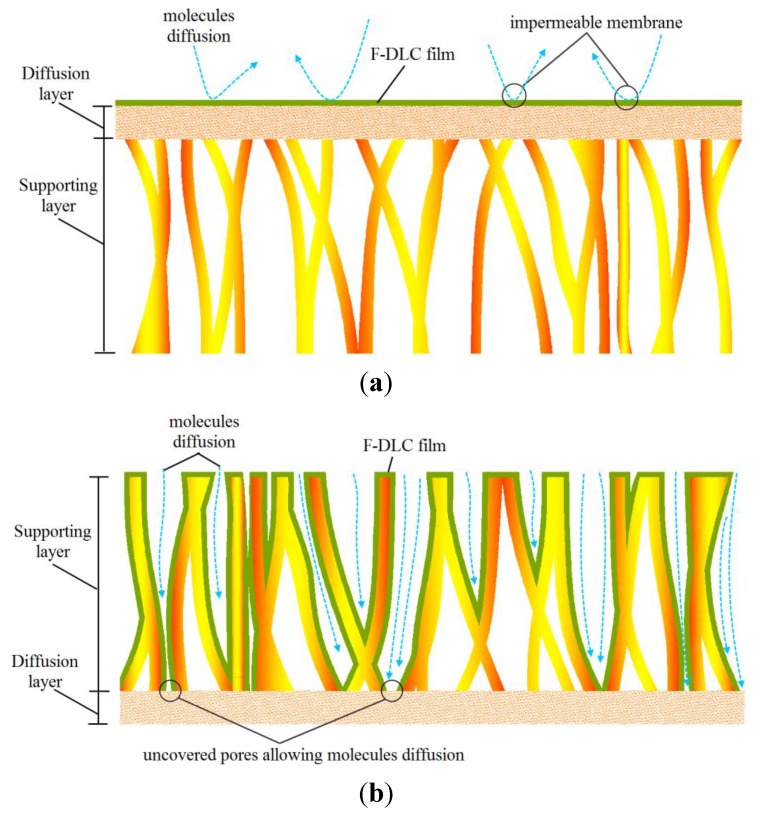
Characteristic of F-DLC film: (**a**) deposited on the diffusion layer; (**b**) deposited on the finger like structure layer.

The diffusivity of electrolyte (K^+^) is an important parameter in the separation membrane evaluation. Figure 8 shows the diffusion coefficient of diffusion layer and finger like structure layer of the PES membrane coated by F-DLC films. As shown in Figure 8, the diffusion coefficient of the diffusion layer and finger like structure layer of bare PES membrane give similar diffusion coefficient value. This means any small molecule can diffuse through the side of the PES membrane, either the diffusion layer or finger like structure layer, and the result remain the same. However, when the diffusion layer of membrane was coated by F-DLC film (F-DLC film deposition time is in the range of 3–6 s), the membrane became impermeable since the F-DLC film blocked the membrane pores, as illustrated in Figure 9a.

On the other hand, when the F-DLC film was coated on the finger like structure layer, the membrane remained permeable despite the fact that the permeability of the membrane is decreased with the increased of the deposited F-DLC film. This is due to the conformal-type of coating of the F-DLC film deposited on the inner wall of the finger like structure layer, as shown in Figure 9b. Based on those results, only PES membrane and PES membrane coated on the finger like structure layer were used in the next diffusion test (long-term) in this study.

**Figure 10:**
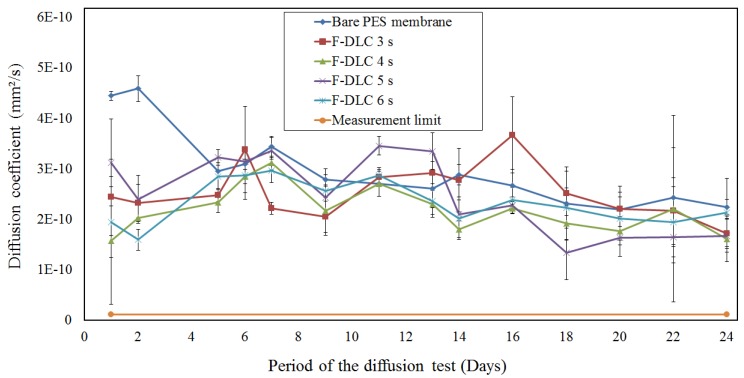
Diffusion coefficient of the membrane for 24 days.

The selected membranes, which were selected based on permeability test result, were then evaluated under long term diffusion test. Figure 10 shows the average diffusion coefficient of the bare PES membrane from the start to the end of 24 days long term diffusion test. As shown in Figure 10, the diffusion coefficient of the bare PES membrane was about 4.4 × 10 mm^2^/s, while those were 2.4 × 10^−^^10^ mm^2^/s, 1.6 × 10^−^^10^ mm^2^/s, 3.1 × 10^−^^10^ mm^2^/s and 1.9 × 10^−^^10^ mm^2^/s for PES membranes coated with F-DLC films with deposition time are 3 s, 4 s, 5 s and 6 s, respectively. Bare PES membranes gave the highest diffusion coefficient compared to other coated membranes, due to the absence of F-DLC film on its surface. The diffusion coefficient of the coated PES membrane appeared to be unpredicted, where the diffusion coefficient of the membrane should be decreased with the increased of the F-DLC film deposition time. The morphology of the finger like structure in the PES membrane has made the F-DLC films unable to be deposited properly on the inner wall of the tunnels structure. Moreover, the range of deposition time among those F-DLC films differed only about 1–3 s. Therefore, a longer deposition time in some way has less effect to the diffusion coefficient of the membrane, which made diffusion coefficient of 5 s of F-DLC deposition time was higher than 3 s and 4 s of F-DLC deposition time. 

As can also be seen from Figure 10, after 7–10 days of the diffusion test, the diffusivity of K^+^ ion through the PES membrane was significantly decreased. When a PES membrane is introduced for long term blood diffusion, adhesion of protein and blood cells may occur. This adhesion will bring forth a fibrous tissue formation, which covers the diffusion area and causing a membrane fouling, as shown in Figure 7. The tissue formation started with blood cell sticking on the membrane surface on day 1 and the membrane coverage intensively increased after several days of diffusion test [17]. The PES membrane fouling increased significantly from day 0 to day 5, and then remained stable from day 5 to day 24. The fact that the molecule diffusion significantly decreased in the first five days and remained consistent after day 5 of the diffusion test can be explained by the fact that the membrane fouling occurred shortly after the membrane contact with blood. The fibrous tissue formation grew thicker within the period of the diffusion test and reached the thickest at the end of the test (day 24). Due to the porosity of the tissue, fibrous tissue on the membrane surface still allowed some nano molecules to diffuse [18]. Therefore, the diffusion coefficient of the membrane remained stable between day 5 and day 24 of the diffusion test.

Figure 10 also shows that the PES membranes coated with F-DLC films have relatively constant diffusion coefficient value. This should be attributed to antithrombogenicity properties of F-DLC film, which had been coated on the membrane surface as well as on the inner wall of the finger like structure porous. Based on some studies, the antithrombogenicity of the F-DLC film improves the blood compatibility of the PES membrane by preventing the platelet adhesion and blood cell absorption [10].

The diffusion test for all the membranes was then continued for 24 days, where diffusion coefficient values of all membranes showing the same decreasing pattern. However, on day 24, the PES membrane had the highest diffusion coefficient (2.2 × 10^−^^10^ mm^2^/s) compared to other tested membranes, and followed by 6 s, 3 s, 5 s and 4 s of F-DLC films deposition times. It can be explained that the number of biomaterials, which are blood coagulation, platelets and proteins are increasing with the duration of diffusion test. Those accumulated biomaterials clogged the membrane porous resulting in the reduction of diffusion coefficient.

## 4. Conclusions

The purpose of this study was to improve the performance of PES membrane in blood compatibility without losing its permeability. F-DLC film was coated on the finger like structure layer of the PES membrane surface and deposited on the inner wall of the finger like structure layer. This deposition technique has successfully had the F-DLC film on the PES membrane without clogging the porous. The contact angle measurement has confirmed the presence of F-DLC film on the membrane surface. The long term diffusion test verified that the permeability of PES membrane significantly dropped in the first 10 days. Whilst, the membrane coated with F-DLC film relatively showed a consistent value of diffusion coefficient. In addition, the blood compatibility of the membrane improved after F-DLC coating, shown by almost no blood cell adhered on the membrane surface on day 1 of the diffusion test. However, after 24 days of the diffusion test, blood clots appear on the bare PES membrane and modified PES membrane. This probably due to the poor adhesion of F-DLC films on the PES membrane. In the future, a different parameter of the F-DLC deposition process such as radio frequency power and fluorine ratio and chemical and physical cleaning of PES membrane prior to the F-DLC deposition will be observed to find a more reliable F-DLC film to be used under long term diffusion test.

## References

[1] Su B., Sun S., Zhao C. (2011). Progress in Hemodialysis—From Emergent Biotechnology to Clinical Practice.

[2] Zhao C.S., Liu T., Lu Z.P., Cheng L.P., Huang J. (2001). An evaluation of a polyethersulfone hollow fiber plasma separator by animal experiment. Artif. Organs.

[3] Tullis R.H., Duffin R.P., Zech M., Ambrus J.L. (2002). Affinity hemodialysis for antiviral therapy. I. Removal of HIV-1 from cell culture supernatants, plasma, and blood. Ther. Apher..

[4] Samtleben W., Dengler C., Reinhardt B., Nothdurft A., Lemke H.D. (2003). Comparison of the new polyethersulfone high-flux membrane DIAPES HF800 with conventional high-flux membranes during on-line haemodiafiltration. Nephrol. Dial. Transplant..

[5] Werner C., Jacobasch H.J., Reichelt G. (1995). Surface characterization of hemodialysis membranes based on streaming potential measurements. J. Biomater. Sci. Polym..

[6] Liu Z., Deng X., Wang M., Chen J., Zhang A., Gu Z., Zhao C. (2009). BSA-modified polyethersulfone membrane: Preparation, characterization and biocompatibility. J. Biomater. Sci. Polym..

[7] Higuchi A., Shirano K., Harashima M., Yoon B.O., Hara M., Hattori M., Imamura K. (2004). Chemically modified polysulfone hollow fibers with vinylpyrrolidone having improved blood compatibility. Biomaterials.

[8] Prihandana G.S., Ito H., Nishinaka Y., Kanno Y., Miki N. (2012). Polyethersulfone membrane coated with nanoporous Parylene for ultrafiltration. J. Microelectromech. Syst..

[9] Asakawa R., Nagashima S., Nakamura Y., Hasebe T., Suzuki T., Hotta A. (2011). Combining polymers with diamond-like carbon (DLC) for highly functionalized materials. Surf. Coat. Technol..

[10] Saito T., Hasebe T., Yohena S., Matsuoka Y., Kamijo A., Takahashi K., Suzuki T. (2005). Antithrombogenicity of fluorinated diamond-like carbon films. Diam. Relat. Mater..

[11] Alsari A.M., Khulbe K.C., Matsuura T. (2001). The effect of soldium dodecyl sulfate solutions as gelation media on the formation of PES membranes. J. Membr. Sci..

[12] Gu Y., Miki N. (2007). A microfilter utilizing a polyethersulfone porous membrane with nanopores. J. Micromech. Microeng..

[13] Gu Y., Miki N. (2009). Multilayered microfilter using a nanoporous PES membrane and applicable as the dialyzer of a wearable artificial kidney. J. Micromech. Microeng..

[14] Kirschbaum B. (2003). The effect of hemodialysis on electrolytes and acid-base parameters. Clin. Chim. Acta.

[15] Morgera S., Haase M., Ruckert M., Krieg H., Kastrup M., Krausch D., Vargas-Hein O., Zuckermann-Becker H., Peters H., Pohlmeier R. (2005). Regional citrate anticoagulation in continuous hemodialysis-acid-base and electrolyte balance at an increased dose of dialysis. Nephron. Clin. Pract..

[16] Boom R.M., Wienk I.M., van den Boomgaard Th., Smolders C.A. (1992). Microstructures in phase inversion membranes. Part 2. The role of a polymeric additive. J. Membr. Sci..

[17] Anderson J.M., Rodriguez A., Chang D.T. (2008). Foreign body reaction to biomaterials. Semin. Immunol..

[18] Wisniewski N., Klitzman B., Miller B., Reichert W.M. (2001). Decreased analyte transport through implanted membranes: Differentiation of biofouling from tissue effects. J. Biomed. Mater. Res..

